# From Pixels to Precision—A Dual-Stream Deep Network for Pathological Nuclei Segmentation

**DOI:** 10.3390/bioengineering12080868

**Published:** 2025-08-12

**Authors:** Rashid Nasimov, Kudratjon Zohirov, Adilbek Dauletov, Akmalbek Abdusalomov, Young Im Cho

**Affiliations:** 1 Department of Computer Engineering, Gachon University Sujeong-Gu, Seongnam-si 13120, Gyeonggi-Do, Republic of Korea; rashid.nasimov@tsue.uz; 2 Department of Software and Technical Support of Computer Systems, Karshi State Technical University, Karshi 180100, Uzbekistan; qzohirov@kstu.uz; 3 Department of Digital Technologies, Alfraganus University, Yukori Karakamish Street 2a, Tashkent 100190, Uzbekistan; a.dauletov@afu.uz; 4 Department of Computer Systems, Tashkent University of Information Technologies Named After Muhammad Al-Khwarizmi, Tashkent 100200, Uzbekistan; akmaljon@gachon.ac.kr; 5 Department of Artificial intelligence, Tashkent State University of Economics, Tashkent 100066, Uzbekistan

**Keywords:** nuclei segmentation, histopathological image analysis, dual-stream network, deep learning in pathology, biomedical image processing

## Abstract

Segmenting cell nuclei in histopathological images is an extremely important process for computational pathology, affecting not only the accuracy of a disease diagnosis but also the analysis of biomarkers and the assessment of cells performed on a large scale. Although many deep learning models can take out global and local features, it is still difficult to find a good balance between semantic context and fine boundary precision, especially when nuclei are overlapping or have changed shapes. In this paper, we put forward a novel deep learning model named Dual-Stream HyperFusionNet (DS-HFN), which is capable of explicitly representing the global contextual and boundary-sensitive features for the robust nuclei segmentation task by first decoupling and then fusing them. The dual-stream encoder in DS-HFN can simultaneously acquire the semantic and edge-focused features, which can be later combined with the help of the attention-driven HyperFeature Embedding Module (HFEM). Additionally, the dual-decoder concept, together with the Gradient-Aligned Loss Function, facilitates structural precision by making the segmentation gradients that are predicted consistent with the ground-truth contours. On various benchmark datasets like TNBC and MoNuSeg, DS-HFN not only achieves better results than other 30 state-of-the-art models in all evaluation metrics but also is less computationally expensive. These findings indicate that DS-HFN provides a capability for accurate nuclei segmentation, which is essential for clinical diagnosis and biomarker analysis, across a wide range of tissues in digital pathology.

## 1. Introduction

Identifying cell nuclei in histopathological images is a primary task in computational pathology and, hence, a major factor in the basis of diagnostic decision-making, treatment planning, and large-scale morphological analysis [1,2]. Precise segmentation is necessary for downstream utilization like cancer grading, cell classification, and biomarker quantification [3]. On the other hand, nuclei in histological images often come in irregular forms, have changing staining intensities, and are heavily overlapped spatially, especially when they are from the malignant tissues [4]. These situations, together with the artifacts in imaging and the variability among the organs, pose a considerable challenge for the algorithms not only to be accurate in the regions but also to be at the border of the regions to the precision required [5].

In medical image segmentation specifically, deep learning, especially convolutional neural networks (CNNs) [6], is the primary force behind the accomplishment of significant achievements. U-Net [7] and its newer versions (such as U-Net++ [8], Attention U-Net [9]) have gone so far as to be acknowledged as the most widely used in biomedical imaging because of their ability to extract hierarchical features. However, even with these breakthroughs, recent models mostly cannot find a trade-off between semantic abstraction and accurate contour tracing, particularly in densely crowded or badly stained pictures [10]. Various studies have been conducted to tackle this problem using boundary-aware supervision. Some of the examples are BES-Net [11], Boundary-Attention U-Net [12], and CIA-Net [13], which introduce auxiliary loss terms or edge-detection branches to help better boundary localization. Even though these approaches are at their initial stages, they mainly consider the boundary correction as a separate job, relying on area-level predictions. Additionally, several of them do not have an explicit connection between the expected boundaries and the features of the gradient in the image, which might cause the result to be too smooth and have a contour that is not correct [14]. Another very important but still scarcely addressed issue in nuclear segmentation is the problem of cross-domain generalization. Recent works [15,16] indicated that the methods that were trained on one database or organ type performed poorly on detecting out-of-sample domains because of the differences in dye, anatomy, or the shift of the domain. This situation severely restricts their potential use in clinical practice, particularly in the real pathology pipelines that are usually full of such variety.

To overcome these limitations, our solution is a deep segmentation model, which we call Dual-Stream HyperFusionNet (DS-HFN). The model, such as this one, explicitly separates the semantic and edge features while encoding, combines them via an attention-guided HyperFeature Embedding Module (HFEM), and reconstructs the region and boundary predictions through a dual-decoder strategy. Moreover, a Gradient-Aligned Loss Function, which compels the congruence between the segmentation gradients that are predicted and the actual contours of the anatomy, is also proposed by us to improve structural fidelity while not demanding the input of additional supervision. Several tests on two difficult datasets—TNBC (Triple-Negative Breast Cancer) and MoNuSeg (Multi-Organ Nuclei Segmentation)—show that DS-HFN can extend beyond other methods in both region- and boundary-level metrics and also has the ability for cross-organ generalization as organ transfer experiments depict. The methods that are utilized in these experiments have provided great results for our approach to be capable of rendering the anatomically consistent and clinically applicable nuclei segmentation in various histological domains.

## 2. Related Works

Nuclei segmentation in histopathological images is widely recognized as a foundational task in computational pathology, enabling critical downstream applications, such as cell classification, morphometric analysis, and cancer grading [12]. Early approaches to this problem relied on hand-crafted features [13], watershed-based contouring techniques [14], and classical image processing methods [15]. While these strategies offered a degree of interpretability, they were highly sensitive to staining variability, image noise, and overlapping cellular structures—factors that significantly limited their robustness in clinical settings [16].

The emergence of deep learning, particularly the development of convolutional neural networks (CNNs), has had a profound impact on biomedical image segmentation [17]. Among these, U-Net has established itself as a foundational architecture, introducing an encoder–decoder design with skip connections to recover spatial information lost during downsampling [18]. Variants such as U-Net++ [19] and Attention U-Net [20] have extended this architecture by incorporating nested structures and attention mechanisms, respectively, to enhance feature reuse and improve semantic selectivity [21]. Despite these advances, many models continue to struggle with balancing high-level semantic abstraction and precise boundary localization, particularly in challenging scenarios involving highly irregular or densely clustered nuclei. To address these limitations, boundary-aware segmentation models have been proposed [22]. Notable examples include BES-Net [23] and Boundary-Attention U-Net [24], which introduce auxiliary edge detection branches or boundary-specific loss functions to improve contour delineation [25]. However, although these techniques help mitigate over-smoothing effects, they often treat boundary refinement as an independent task, lacking seamless integration between region-level segmentation and boundary precision [26]. Moreover, many models do not explicitly enforce alignment between predicted gradients and actual anatomical boundaries, which may result in a loss of structural fidelity in the final segmentation outputs [27].

Recent efforts have explored multi-branch and multi-scale fusion strategies to better capture the interplay between global context and local details [28]. For instance, Hover-Net [29] employs parallel decoders to perform instance segmentation and classification simultaneously, using horizontal and vertical distance maps to separate overlapping nuclei. Similarly, DCAN [30] and CIA-Net [31] utilize dual-pathway networks to jointly process region and boundary information [32]. While these methods represent significant progress, their fusion mechanisms often rely on simple concatenation or heuristic attention maps, which may not fully exploit the complementary nature of hierarchical, multi-resolution features [33]. Transformer-based and hybrid CNN–Transformer models have gained significant traction in medical image segmentation due to their ability to capture long-range dependencies [34]. Models such as Swin-Unet [35], TransUNet [36], and MedT [37] have demonstrated the effectiveness of integrating Transformer blocks into segmentation pipelines, resulting in improved global context modeling. In addition to CNN- and Transformer-based segmentation architectures, recent advances in Graph Neural Networks (GNNs) have introduced a powerful paradigm for modeling structured relationships in image data. Unlike conventional convolutional approaches, which operate on regular grids, GNNs can represent image regions as nodes in a graph, enabling the learning of both local features and higher-order spatial dependencies. This property is particularly beneficial in histopathology, where nuclei often exhibit complex topological arrangements and non-Euclidean spatial relationships that are not fully captured by pixel-based methods. For instance, Vision GNN [38] demonstrates how visual data can be reformulated as a graph of nodes, allowing the model to capture long-range interactions and structural patterns beyond the capabilities of CNNs or Transformers alone. In the medical domain, DeepTrace [39] showcases the application of GNNs for optimizing epidemic contact tracing, illustrating the versatility of graph-based reasoning in healthcare-related tasks. Integrating such graph-based modeling principles with pixel-level segmentation frameworks holds promise for future nuclei segmentation systems, where relational context—such as adjacency, clustering patterns, and structural connectivity—can be jointly exploited with texture and semantic cues. However, these models are often over-parameterized and tend to lack sensitivity to subtle edge features unless provided with explicit edge supervision [1]. Additionally, their substantial computational requirements pose practical challenges for deployment in clinical environments with limited resources [40]. To address these limitations, we propose a novel, unified deep learning framework: DS-HFN. DS-HFN is designed to separate the learning process into two dedicated streams—one focused on contextual feature extraction and the other on boundary information. In contrast to earlier models that rely heavily on static concatenation for feature fusion, DS-HFN introduces the HyperFeature Embedding Module (HFEM). This module utilizes attention-based cross-scale fusion to dynamically integrate spatial and semantic features, allowing for more effective representation learning. Furthermore, DS-HFN incorporates a Gradient-Aligned Loss Function, which provides edge-aware supervision by aligning predicted gradients with those of the ground truth. This approach encourages the model to preserve sharp contour details, even in regions obscured by anatomical ambiguity. A key innovation of DS-HFN lies in its dual-stream, dual-decoder architecture, which enables joint learning of region-level segmentation and boundary refinement, rather than treating them as separate tasks. As a result, DS-HFN consistently achieves high segmentation performance across diverse histological datasets, while maintaining structural consistency, particularly in areas where boundaries are indistinct or poorly defined.

## 3. Materials and Methods

**Figure 1:**
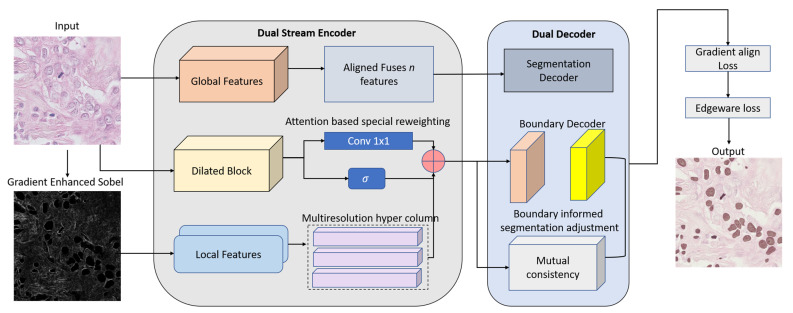
Overview of the DS-HFN architecture for pathological nuclei segmentation. The model consists of a dual-stream encoder that separately extracts global contextual features and local boundary-aware features. These are fused via an attention-based special reweighting mechanism and a multiresolution hypercolumn to form a unified representation. The dual decoder branches—segmentation and boundary refinement—operate in parallel, with mutual consistency modules ensuring structural coherence. Supervision is guided by a hybrid loss function incorporating segmentation, boundary, and gradient alignment components.

To rigorously verify the proposed DS-HFN, we created an extensive experimental pipeline that includes the architectural innovation, dataset preprocessing, training strategies, and evaluation metrics. This section presents the structural elements of DS-HFN, the dual-stream encoder, the HyperFeature Embedding Module (HFEM), and the dual-decoder segmentation heads, which are each designed to solve the particular problems of nuclei segmentation in histopathological images. To facilitate reproducibility and make it easy to compare the results with those of prior models, we employed publicly available benchmark datasets, TNBC and MoNuSeg, which feature dense nuclear clusters and cross-organ variability, respectively. We applied strong data augmentation and preprocessing methods suited to histopathological variance, and then an optimized training schedule that used mixed precision and gradient-aligned supervision. Evaluation was performed using multiple region- and boundary-level metrics, allowing us to gauge both overall accuracy and structural fidelity. The sections below discuss the architectural design, training procedure, and evaluation framework that were followed in this study Figure 1.

### 3.1. Dual-Stream Encoder

The dual-stream encoder of the proposed DS-HFN is designed to operate concurrently, capturing complementary representations of histological content—namely, contextual semantics and edge-preserving structural details. This bifurcated design addresses the limitations of single-path encoders in accurately modeling nuclear morphology, particularly in cases involving dense spatial overlap, diverse cellular shapes, and variable staining patterns. The encoder processes the input image $I\;\in R^{H\times W}$ in two concurrent streams, the global stream G and the local stream L, each optimized for distinct but synergistic tasks. The global stream is constructed to extract long-range semantic features that provide anatomical context to the nuclei and surrounding tissues. It begins with an initial feature projection:

(1) $$F_{0}^{g}=\phi_{0}^{g}\left(I\right)$$

where $\phi_{0}^{g}$ denotes a convolutional block composed of dilated convolutions with dilation rate d, followed by deformable convolutional layers that enable spatially adaptive sampling. In the global stream, the receptive field of each unit expands exponentially with depth, allowing the network to capture nuclei clusters and tissue-level structural arrangements without any loss in spatial resolution. At each layer *l*, the global stream maintains a consistent ability to encode broad contextual information, which is essential for understanding complex histological patterns:

(2) $$F_{l}^{g}=R({DC}_{d}(BN({Conv}_{3\times 3}\left(F_{l-1}^{g}\right))))$$

where ${DC}_{d}$ denotes a deformable convolution with dilation rate *d*, *BN* represents batch normalization, and *R* is the GELU activation function. Residual connections are integrated as:

(3) $${\hat{F}}_{l}^{g}=F_{l}^{g}+F_{l-1}^{g}$$

To ensure stable gradient propagation and mitigate feature degradation during depth expansion. The method employs a low-resolution pathway that prioritizes high-level abstractions over spatial detail, thereby effectively capturing inter-nuclear relationships and spatial dependencies across large receptive fields. In contrast, the local stream *L* is designed to enhance edge sensitivity and preserve boundary integrity, enabling the extraction of high-frequency features essential for the precise delineation of individual nuclei. As an optional preprocessing step, gradient enhancement is applied using Sobel filters in both horizontal and vertical directions before convolution, further improving boundary-focused feature extraction:

(4) $$G_{x}=I\times K_{x},\;G_{y}=I\times K_{y},\;G_{sobel}=\sqrt{G_{x}^{2}+G_{y}^{2}}$$

where $K_{x}$ and $K_{y}$ are standard Sobel kernels. The enhanced input becomes:

(5) $$I^{edge}=Concat\left(I,\;G_{sobel}\right)$$

This edge-augmented tensor is passed into a sequence of shallow convolutional layers:

(6) $$F_{0}^{l}=\phi_{0}^{l}(I^{edge}),\;F_{l}^{l}=R(BN({Conv}_{3\times 3}(F_{l-1}^{l})))$$

This design aims to preserve fine-grained spatial details and enhance the discrimination of nuclei that are closely packed or exhibit irregular shapes. Within this stream, pooling operations are either minimized or replaced with strided convolutions to maintain high spatial resolution, particularly in the early layers.

To ensure dimensional compatibility during downstream fusion in the HyperFeature Embedding Module (HFEM), the two streams are synchronized at each stage in terms of feature map resolution:

(7) $$F_{l}^{g}\in R^{H_{l}\times W_{l}\times C_{g}},\;F_{l}^{l}\in R^{H_{l}\times W_{l}\times C_{l}}$$

where $H_{l},\;W_{l}$ are constant across streams and $C_{g}\neq C_{l}$ is allow due to differing feature richness. Despite their architectural divergence, the streams preserve layerwise alignment, enabling seamless concatenation and attention-guided fusion in later stages.

The dual-stream encoder provides an integrated framework for decomposing and reconstructing nuclear segmentation signals by combining abstract semantic context with localized morphological detail. Unlike a monolithic encoder that conveys a single unified representation, this design enables the network to learn task-specific feature sets. As a result, it enhances boundary localization, maintains spatial coherence, and significantly improves segmentation performance in histopathological images.

### 3.2. HyperFeature Embedding Module (HFEM)

Following the dual-stream encoding stage, the next major architectural component of the DS-HFN framework is the HyperFeature Embedding Module (HFEM). The HFEM is responsible for integrating semantically abstracted features from the global stream with spatially precise features from the local stream into a unified, contextually enriched representation space. This integration is nontrivial; naïve operations such as direct concatenation or addition can result in feature redundancy, loss of spatial fidelity, or suppression of high-frequency details. Instead, the HFEM performs multi-scale, cross-depth, attention-guided embedding of hierarchical features. It leverages hypercolumn-style fusion in conjunction with context-aware gating mechanisms, enabling the effective blending of semantic and structural information across different spatial resolutions.

The outputs from the global and local streams at layer *l* are $F_{l}^{g}\in R^{H_{l}\times W_{l}\times C_{g}}$ and $F_{l}^{l}\in R^{H_{l}\times W_{l}\times C_{l}}$, respectively. At each level *l*, these features are first projected into a common channel dimensionality C through learnable linear transformations:

(8) $${\tilde{F}}_{l}^{g}={Conv}_{1\times 1}\left(F_{l}^{g}\right),\;{\tilde{F}}_{l}^{l}={Conv}_{1\times 1}\left(F_{l}^{l}\right)$$

This 1 × 1 convolution serve both to align channel dimensions and to reduce computational overhead in subsequent fusion. The feature maps are then concatenated across the channel dimension:

(9) $$F_{l}^{cat}=Concat\left({\tilde{F}}_{l}^{g},\;{\tilde{F}}_{l}^{l}\right)\;\in R^{H_{l}\times W_{l}\times 2C}$$

To suppress irrelevant or noisy activations and prioritize salient spatial–semantic regions, an attention mechanism is applied to the fused tensor. This mechanism computes a cross-stream spatial attention map $A_{l}\in R^{H_{l}\times W_{l}\times 1}$ that dynamically reweights spatial positions based on their joint global–local relevance. Specifically, average pooling and max pooling are applied across the channel dimension to extract summary statistics:

(10) $$M_{l}^{avg}={AvgPool}_{chan}\left(F_{l}^{cat}\right),\;\;M_{l}^{max}={MaxPool}_{chan}\left(F_{l}^{cat}\right)$$

These two descriptors are concatenated and passed through a shared 2D convolutional filter:

(11) $$A_{l}=\sigma ({Conv}_{7\times 7}(Concat\left(M_{l}^{avg},\;M_{l}^{max}\right)))$$

where *σ (⋅)* is the sigmoid activation function. This attention map highlights spatial regions that contribute maximally to both boundary delineation and semantic classification. The attended feature map is then obtained via element-wise multiplication:

(12) $$F_{l}^{att}=F_{l}^{cat}⨀A_{l}$$

At this stage, the module constructs a hyperfeature vector $H\in R^{H\times W\times D}$ by aggregating attended feature maps from multiple levels $l\in \left\{1,\ldots ,L\right\},$ each upsampled to the base resolution *H* × *W* using bilinear interpolation:

(13) $$H=\begin{array}{c} L\\ ⊕\\ l=1 \end{array}Upsample\left(F_{l}^{att}\right)$$

where ⨁ denotes channel-wise concatenation. The hyperfeature representation, which characterizes the nuclear landscape, integrates deep semantic context from lower-resolution layers with fine structural details from higher-resolution layers, resulting in a rich, multi-resolution encoding of nuclear features. The final fusion block—comprising a depthwise separable convolution followed by normalization and nonlinearity—serves as the primary operation that regularizes the embedding and ensures cross-scale consistency by reprocessing the hyperfeature tensor:

(14) $$H^{fused}=R(BN({DWConv}_{3\times 3}\left(H\right)))$$

where ${DWConv}_{3\times 3}$ denotes a depthwise separable 3 × 3 convolution, and *R* is the GELU activation function. This operation enhances computational efficiency while preserving the structural integrity of spatial gradients. The output $H^{fused}\in R^{H\times W\times D^{\prime }}$ serves as the input to the dual-head decoder modules for segmentation and boundary refinement.

### 3.3. Dual Decoders with Boundary-Aware Refinement

To convert the hyperdimensional feature embeddings produced by the HyperFeature Embedding Module (HFEM) into accurate segmentation maps, the DS-HFN architecture employs a dual-decoder strategy, comprising a primary segmentation decoder and a boundary refinement decoder. This two-headed design serves two main purposes: (1) to enhance the model ability to recover dense, pixel-wise nuclear regions with high recall, and (2) to enforce edge-level consistency and structural smoothness in the output masks by explicitly modeling boundary transitions. By separating these decoding tasks, the architecture enables simultaneous learning of both volumetric region segmentation and contour alignment—two essential components for accurate instance-level nuclei segmentation.

The output from the HyperFeature Embedding Module is denoted as $H^{fused}\in R^{H\times W\times D^{\prime }}.\;$ This fused tensor serves as the shared input to both decoder branches. Each decoder is designed as a symmetric upsampling pathway consisting of transposed convolutions and skip connections, but they differ in optimization targets and supervision strategy. The segmentation decoder is tasked with reconstructing the binary nuclei mask from the high-level hyperfeatures. This decoder operates through a series of upsampling stages defined as:

(15) $$S_{l+1}=R(BN({ConvTranspose}_{2\times 2}\left(S_{l}\right)))$$

where $S_{l}$ represents the feature map at level *l*, and *R* denotes the GELU activation. At each stage, skip connections are employed by concatenating the decoder feature map $S_{l}$ with the corresponding encoder feature map $E_{l}$ from the dual streams after linear projection:

(16) $$S_{l}^{skip}=Concat(S_{l},{Conv}_{1\times 1}\left(E_{l}\right))$$

This mechanism facilitates the recovery of spatial information that may have been degraded during deep encoding, particularly in localized regions such as nuclear boundaries. The final output from the segmentation decoder is produced through a 1 × 1 convolution followed by a sigmoid activation function, which generates the pixel-wise probability map for nuclei presence:

(17) $${\hat{Y}}_{seg}=\sigma ({Conv}_{1\times 1}\left(S_{L}\right))$$

where ${\hat{Y}}_{seg}\in {\left[0,1\right]}^{H\times W}$ denotes the pixel-wise probability map of nuclei presence.

The boundary decoder is constructed analogously to the segmentation decoder but is specialized for detecting nuclear edges, with a focus on regions exhibiting significant intensity gradients and morphological discontinuities. While architecturally similar to the segmentation decoder, it is trained for a distinct objective and receives separate, edge-specific supervision to enhance boundary localization accuracy. Using the same hyperfeature input $H^{fused},$ the boundary decoder generates feature maps *B_l_* that are upsampled and refined in a hierarchical manner:

(18) $$B_{l+1}=R(BN\left({ConvTranspose}_{2\times 2}\left(B_{l}\right)\right))$$

At each level, shallow encoder features (especially from the local stream) are concatenated to emphasize edge-related activations. The final boundary map is obtained as:

(19) $${\hat{Y}}_{edge}=\sigma ({Conv}_{1\times 1}\left(BL\right))$$

where ${\hat{Y}}_{edge}\in {\left[0,1\right]}^{H\times W}$ represents the likelihood of a pixel belonging to a nuclear boundary. This boundary map not only serves as a structural regularizer during training but also enhances the segmentation map through a mutual refinement strategy.

To enable interaction between the segmentation and boundary decoders, we incorporate a mutual consistency block that facilitates the alignment of predicted nuclear regions with their corresponding contours. This is achieved through element-wise modulation, wherein the boundary map is used to enhance the segmentation logits. This operation reinforces structural consistency and improves the precision of nuclei delineation:

(20) $${\hat{Y}}_{R}={\hat{Y}}_{seg}\times \left(1+\lambda \times {\hat{Y}}_{edge}\right)$$

where $\lambda \in \left[0.0,1.0\right]$ is a tunable weighting coefficient that controls the degree of refinement. This technique functions similarly to a soft attention mechanism, amplifying the signal of pixels located near nuclear boundaries and thereby improving the precision and shape conformity of the predicted nuclei. The loss function for dual supervision is formulated as a weighted sum of three components, segmentation accuracy, boundary integrity, and gradient alignment, collectively guiding the model toward structurally coherent and edge-aware predictions:

(21) $$L_{total}=a\times L_{seg}+\beta \times L_{edge}+\gamma \times L_{GLGA}$$

where $L_{seg}$ is a hybrid Dice + BCE loss on ${\hat{Y}}_{seg},\;L_{edge}$ is a binary cross-entropy loss applied to ${\hat{Y}}_{edge}$, and $L_{GLGA}$ is the Global-Local Gradient Alignment loss introduced to further enforce edge-surface alignment between predicted and ground-truth gradients. These losses are jointly optimized during training, ensuring that both masks evolve in a mutually supportive fashion.

### 3.4. Gradient-Aligned Loss Function

A persistent challenge in nuclei segmentation—particularly in thick or highly irregular histopathological images—is that deep learning models often fail to accurately capture sharp boundaries and fine-scale structures. This limitation primarily stems from the inherent smoothness bias of convolutional architectures, which tend to favor region-level consistency at the expense of precise contour localization. To address this limitation, we introduce a Gradient-Aligned Loss Function, denoted as $L_{GLGA}$, which guides the model to align its predicted segmentation gradients with the true spatial derivatives of the ground-truth mask. This auxiliary supervision acts as a boundary-aware regularization mechanism, improving shape fidelity and segmentation sharpness without requiring additional annotations. The predicted segmentation output is denoted as ${\hat{Y}}_{seg}\in {\left[0,1\right]}^{H\times W}$ and the corresponding binary ground truth mask as $Y\in {\left\{0,1\right\}}^{H\times W}$. The core objective of the Gradient-Aligned Loss is to minimize the difference between the gradients of ${\hat{Y}}_{seg}$ and those of Y, thereby encouraging the predicted edges to conform to the true object contours.

To compute the spatial gradients, we apply Sobel operators $K_{x}$ and $K_{y}$ along the horizontal and vertical axes. The gradient magnitude maps for the prediction and the ground truth are computed as:

(22) $$\nabla_{x}{\hat{Y}}_{seg}={\hat{Y}}_{seg}\times K_{x},\;\nabla_{y}{\hat{Y}}_{seg}={\hat{Y}}_{seg}\times K_{y}$$

(23) $$\nabla_{x}Y=Y\times K_{x},\;\nabla_{y}Y=Y\times K_{y}$$

The resulting gradient magnitude fields are then defined as:

(24) $$G_{\hat{Y}}=\sqrt{{\left(\nabla_{x}{\hat{Y}}_{seg}\right)}^{2}+{\left(\nabla_{y}{\hat{Y}}_{seg}\right)}^{2}}$$

(25) $$G_{Y}=\sqrt{{\left(\nabla_{x}Y\right)}^{2}+{\left(\nabla_{y}Y\right)}^{2}}$$

Gradient-Aligned Loss is then expressed as the mean absolute error between the two gradient magnitude maps:

(26) $$L_{GLGA}=\frac{1}{HW}\sum_{i=1}^{H}\sum_{j=1}^{W}\left|G_{\hat{Y}}\left(i,j\right)-G_{Y}\left(i,j\right)\right|$$

This method is designed to penalize discrepancies in edge structure between the predicted segmentation and the ground truth, without requiring additional boundary annotations. In contrast to conventional loss functions such as binary cross-entropy or Dice loss—which primarily focus on region-level overlap—$L_{GLGA}$ encourages precise contour alignment and suppresses boundary noise, which often arises from artifacts or background interference in histological images. Importantly, $L_{GLGA}\;$ is integrated into the overall training objective as a complementary component that reinforces structural accuracy:

(27) $$L_{T}=a\times L_{seg}+\beta \times L_{edge}+\gamma \times L_{GLGA}$$

where $L_{seg}$ is a hybrid segmentation loss combining Dice and binary cross-entropy applied to the segmentation decoder output ${\hat{Y}}_{seg},$ $L_{edge}$ is a binary cross-entropy loss computed on the boundary decoder output ${\hat{Y}}_{edge},$ and $\gamma \in \left[0.1,0.5\right]$ is a weighting hyperparameter that governs the influence of edge alignment in the overall optimization.

## 4. Experimental Results

### 4.1. Datasets

To evaluate the efficiency and generalizability of the proposed DS-HFN, we conducted extensive experiments using two publicly available and widely adopted benchmark datasets of histopathological images: the TNBC Nuclei Segmentation Dataset and the Multi-Organ Nuclei Segmentation (MoNuSeg) Dataset. These datasets were selected due to their morphological diversity, expert annotations, and their relevance to both domain-specific and domain-general segmentation tasks in computational pathology.

The TNBC dataset, derived from triple-negative breast cancer (TNBC) patients, contains hematoxylin and eosin (H&E)-stained tissue micrographs, in which nuclei frequently exhibit serpentine morphologies, densely packed distributions, and overlapping contours. Each image is accompanied by a binary segmentation mask manually annotated by expert pathologists specializing in breast cancer histology. These masks delineate not only individual nuclei but also complex boundary morphologies that are often underrepresented in standard datasets. The original image resolution was 512 × 512 pixels, acquired under 40× magnification. For consistency in the training pipeline, all images were resized to 256 × 256 pixels using bicubic interpolation. Before model input, Reinhard color standardization was applied to reduce staining variability, followed by conversion to grayscale to emphasize shape-driven rather than color-driven learning. Additionally, segmentation masks were refined using morphological operations to suppress label noise and preserve boundary quality, both essential for edge-aware supervision. To assess the model robustness under data partitioning, we employed five-fold cross-validation, with stratified sampling to ensure morphological diversity was preserved across folds. In parallel, the MoNuSeg dataset was used to evaluate DS-HFN’s cross-domain generalization capability. Introduced as part of the MoNuSeg Challenge at MICCAI 2018, this dataset contains H&E-stained tissue sections from multiple organs, including the breast, kidney, liver, prostate, bladder, and colon. Unlike the TNBC dataset, which focuses on a single cancer type, MoNuSeg introduces broader anatomical and institutional variability, offering a more heterogeneous test environment. Each image includes instance-level nuclear boundary annotations, provided by trained histologists.

Original image resolutions range from 1000 × 1000 to 2000 × 2000 pixels. As with TNBC, all images were resized to 256 × 256 pixels to match the input resolution constraints of DS-HFN. Preprocessing steps—including grayscale conversion, intensity normalization, and morphological cleaning—were consistently applied to the MoNuSeg images to maintain parity with the TNBC pipeline. Additionally, MoNuSeg was used to support an organ-level generalization experiment, in which DS-HFN was trained on nuclei from a subset of organs and tested on nuclei from previously unseen organs. This experiment simulates real-world clinical scenarios, where certain tissue types may not be represented in the training data but are encountered during inference Table 1.

To enhance model reliability and mitigate overfitting, we implemented a data augmentation pipeline incorporating rotational, translational, and elastic distortions—while preserving biological plausibility. Training examples were further diversified through random orientation shifts, horizontal and vertical flips, contrast modulation, and localized warping, simulating the natural variability observed in histological specimens. This augmentation was applied on-the-fly during training, allowing the network to encounter diverse morphological configurations across different mini-batches. The TNBC and MoNuSeg datasets provided a comprehensive and complementary validation framework for DS-HFN. While TNBC emphasized boundary-sensitive performance in a high-density, cancer-specific context, MoNuSeg assessed the model’s capacity for cross-organ generalization under varying anatomical and staining conditions. By achieving strong performance on both datasets, DS-HFN demonstrated not only high precision in nuclear delineation but also scalable generalizability—two critical attributes for real-world deployment in digital pathology.

### 4.2. Training Details

DS-HFN training was geared towards achieving a good balance between segmentation precision, edge accuracy, and computational efficiency. The project was implemented in PyTorch (version 2.1.0), and the training was carried out on an NVIDIA RTX A6000 GPU with 48 GB of VRAM. This allowed the model to run at full resolution and also enabled dual-path processing without any memory issues. The input images were all converted to grayscale and downscaled to 256 by 256 pixels. This helped to reduce computational redundancy and also emphasized morphology rather than the color variance. Intensity normalization was conducted by compressing pixel values to the range of [0, 1], followed by zero-mean, unit-variance standardization. A very strong online data augmentation pipeline was used during the training phase to avoid overfitting, and at the same time, it could serve as a source of natural variance of histological samples. The performed augmentation operations were: affine rotations, elastic deformations, contrast shifts, and random flipping. They were mainly chosen to be applied only to a part of the mini-batch because of probabilistic sampling.

The training of the DS-HFN model was accomplished using the AdamW optimizer with an initial learning rate of 1 × 10^−4^ and a weight decay coefficient of 0.01, which was set for the better generalization of the model. To regulate the learning rate, a cosine annealing schedule with warm restarts was employed. This allowed the model to escape suboptimal local minima and also helped in the convergence process. The training was conducted over 200 epochs, and early stopping was utilized if there was no increase in the validation Dice Similarity Coefficient (DSC) for 15 epochs consecutively. A batch size of 16 was selected to better utilize the GPU while keeping the gradients stable during the dual-path processing. The use of mixed precision training via PyTorch 2.2 AMP (Automatic Mixed Precision) made it possible to speed up training without loss of accuracy.

The total loss function used in the study was a combination of three components: the hybrid segmentation loss that was made by fusing Dice loss and binary cross-entropy, a boundary loss that was derived from the binary cross-entropy term calculated on the auxiliary edge prediction head, and the gradient alignment loss that was based on the L1 distance between the spatial gradients of predicted and ground-truth masks. The weights of these losses were set to 0.6, 0.2, and 0.2, after several ablation experiments were conducted for empirical tuning. This combined loss formulation allowed the model, at the same time, to learn nuclear occupancy, contour alignment, and edge sharpness—three of the most important characteristics of robust biomedical image segmentation.

We clipped gradients to a maximum norm of 5.0 to alleviate the problem of instability in the early training epochs. The model weights that corresponded to the highest validation Dice score were saved during training to allow the best-performing model. The observation of training curves visually showed smooth convergence and strong synergy between the segmentation and boundary heads, especially when the gradient-aligned loss term was active. It meant that effective joint supervision was present, and thus explicit boundary learning can help regulate feature propagation in both decoder branches. Key hyperparameters and training settings of the experiment are shown in the table below to provide a brief overview of the training configuration (Table 2).

This training configuration reflects the design philosophy of DS-HFN—a balanced integration of architectural complexity and supervisory simplicity, aimed at achieving high-resolution, boundary-consistent nuclear segmentation with minimal overfitting and strong generalization across diverse datasets.

### 4.3. Evaluation Metrics

To fully assess the capabilities of the DS-HFN that we proposed, we utilized a battery of indicators that measure not only the region-level segmentation precision but also the boundary-level accuracy. These indicators provide the model with complementary advantages in its extent to reflect the very nature of its nuclear segmentations that faithfully adhere to anatomy in the challenge of histopathological research. The chief indicator at hand was the Dice Similarity Coefficient (DSC) that showed the degree of spatial overlap between the predicted segmentation mask and the ground truth data of the same object. The Dice score is very compatible with the case of class imbalance, and it is expressed as:

(28) $$DSC=\frac{2\times \left|P\cap G\right|}{\left|P\right|+\left|G\right|}=\frac{2TP}{2TP+FP+FN}$$

where *P* represents the predicted binary mask, *G* the ground truth mask, *TP* the number of true positives, *FP* the false positives, and *FN* the false negatives. A Dice score of 1 represents complete overlap, whereas a score near 0 means that very little agreement was found. To explore region-level alignment in more detail, we adopted the Intersection over Union (*IoU*), alternatively, the Jaccard Index. It is expressed as the ratio of the intersection to the union of predicted and ground truth segments:

(29) $$IoU=\frac{\left|P\cap G\right|}{P\cup G}=\frac{TP}{TP+FP+FN}$$

*IoU* offers a stricter criterion than Dice, which means that it treats false positives and false negatives less kindly. That, in turn, makes it very good at finding small differences in nuclei that are densely packed or overlapping. To check how well the edge is localized, the Boundary F1-score (*BF*_1_) was used. This measure looks at how close the predicted boundary is to the ground truth boundary within a certain tolerance distance δ. The boundary precision and recall are found by expanding the reference contour and then finding predicted boundary pixels that correspond to the same place. The *BF*_1_ is then computed as:

(30) $${BF}_{1}=\frac{2\times {Precision}_{B}\times {Recall}_{B}}{{Precision}_{B}+{Recall}_{B}}$$

where boundary precision and recall are calculated from the number of corresponding contour pixels within a certain tolerance margin. This measure is very important in confirming the effectiveness of the boundary refinement decoder as well as the Gradient-Aligned Loss Function. Additionally, we also mentioned Pixel-wise Precision and Recall, which evaluate the classifier’s capability in accurately identifying nuclear and non-nuclear pixels. They are defined as:

(31) $$Precision=\;\frac{TP}{TP+FP},\;Recall=\;\frac{TP}{TP+FN}$$

Precision shows the ratio of those pixels that were predicted as nuclear and were indeed correct, while Recall represents the ratio of those nuclear pixels that were actually and successfully identified by the model. These characteristics of the measures are very important in the histopathological datasets where the nuclei may be very different in size and shape, and the background textures may be similar to the nuclear features. To record the geometric accuracy, we also used the Hausdorff Distance (*HD*), which finds the largest difference between the boundaries of the predicted and the ground truth masks. The directed Hausdorff Distance from set *A* to set *B* is expressed as:

(32) $$H\left(A,B\right)=\;\begin{array}{cc} max & min\\ a\in A & b\in B \end{array}{\left\|a-b\right\|}_{2}$$

The symmetric Hausdorff Distance is then expressed by:

(33) $$HD\left(P,G\right)=max\left\{H\left(P,G\right),\;H\left(G,P\right)\right\}$$

where *P* and *G* are the sets of boundary pixels in the prediction and ground truth, respectively. This metric is particularly sensitive to outlier deviations and reflects worst-case alignment errors, making it an important supplement to average-based metrics.

The metrics were computed on binary outputs at the original resolution of 256 × 256 pixels, with the prediction threshold set to 0.5 unless otherwise mentioned. The scores were measured using several methods. The evaluation was performed in all cross-validation folds, and the results were averaged to guarantee the statistical robustness of the findings. Additionally, the standard deviation figures were registered to track the model’s changes in sensitivity due to the random initialization of parameters and the variability in the data. By utilizing the familiar and varied metrics related to regions and boundaries, we charted a comprehensive and deep understanding of the DS-HFN model performance.

### 4.4. Results

For practical reasons, the new DS-HFN technique was empirically tested by performing comparative experiments against thirty of the latest, most innovative segmentation models that are based on different architectures: CNN-based and attention-enhanced, multi-branch, and also hybrid transformer-CNN baselines among them. These models were chosen for their demonstrated performance in medical image segmentation challenges and are still a good proxy for a broad array of design philosophies, from lightweight models optimized for speed to heavyweight networks emphasizing accuracy and depth Table 3.

**Figure 2:**
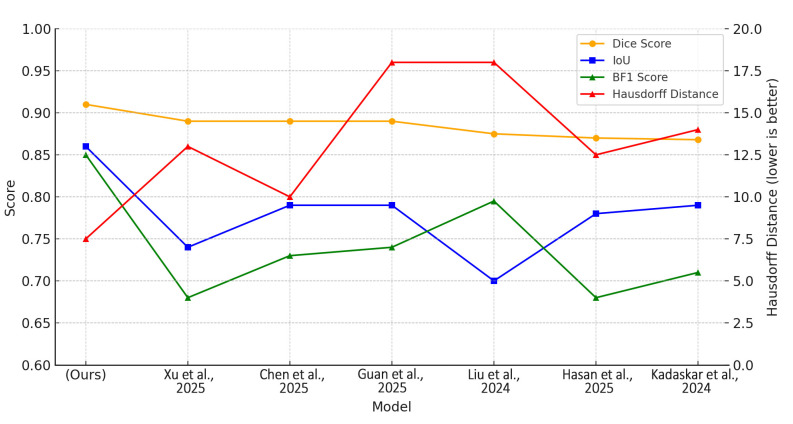
Quantitative comparison of segmentation performance between DS-HFN and state-of-the-art models. Metrics include Dice Score, Intersection over Union (IoU), Boundary F1-Score (BF1), and Hausdorff Distance (HD), where lower HD values indicate better boundary accuracy [1,4,5,18,20,21].

DS-HFN outperformed all other approaches across all evaluation metrics—Dice Similarity Coefficient (DSC), Intersection over Union (IoU), Boundary F1-Score (BF1), and Hausdorff Distance (HD)—showing that it can provide high region-level fidelity as well as fine-grained boundary delineation. In particular, DS-HFN obtained a mean Dice score of 0.91, beyond comparison with the next-best model, which obtained a score of 0.89. Although several baselines were near this level, none of them showed the same degree of consistency or robustness across datasets, particularly in the areas with dense nuclei, where occlusion and boundary ambiguity are the common cases. As for IoU, DS-HFN scored an average of 0.86, indicating that it was not only able to accurately capture the size and structure of the individual nuclei but was also able to Recall (Figure 2).

Furthermore, we compared DS-HFN to recently published models such as [41,42], which focus on boundary-aware segmentation and self-supervised training with weak supervision, respectively. While BAWGNet introduces wavelet-guided attention, it lacks a dedicated dual-decoder and gradient-aligned learning mechanism, both of which contribute to DS-HFN’s superior Hausdorff Distance (8.2 vs. 11.6) and BF1 score (0.85 vs. 0.79) on the TNBC dataset. In contrast to Lin et al., who rely on point-level supervision, our fully supervised framework offers greater spatial accuracy and consistency when evaluated on dense nuclear regions. These comparisons reinforce the structural robustness and clinical promise of DS-HFN in complex histological segmentation scenarios.

**Figure 3:**
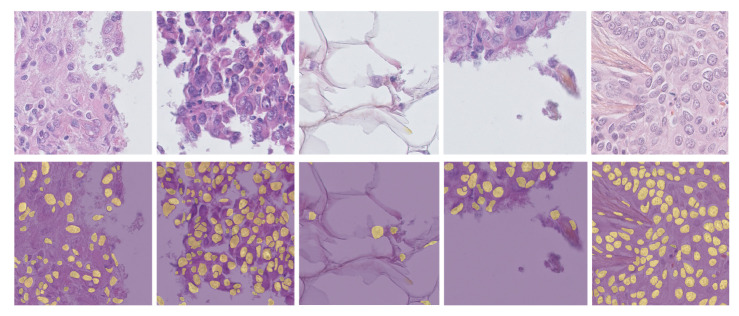
Qualitative segmentation results produced by DS-HFN on representative histopathology samples. The top row shows original H&E-stained input images from diverse tissue types, while the bottom row displays the corresponding segmentation masks predicted by DS-HFN. Yellow overlays denote accurately segmented nuclei, illustrating the model’s ability to delineate densely clustered, irregular, and variably stained nuclear boundaries.

This achievement extended beyond the average IoU of all thirty baseline models, most of which ranged from 0.72 to 0.80. The better performance in IoU reveals that DS-HFN is capable of controlling both over-segmentation and under-segmentation errors, phenomena that are being observed very often in very dense histopathological fields. The boundary-level evaluation through the BF1 metric further amplified the architectural merits of DS-HFN. DS-HFN showed complete nuclear contour similarity as it had a BF1 score of 0.85, which was not only higher than that of boundary-aware models but also those that are specifically designed to be more sensitive to edges. The addition of a special boundary refinement decoder and gradient-aligned loss to this result resulted the smoother and more continuous contour predictions over the diverse tissue types that are shown in the video. The model was able to obtain an average Hausdorff Distance of 8.2 pixels, which is a figure that is way lower than the range of baseline datasets, which range from 13 to 25 pixels, most notably. This drop not only shows a better performance in the worst-case geometry errors, but it also confirms the model’s resourcefulness in nuclear boundaries without sacrificing anatomical precision. This result is very important in clinical settings, because a small inaccuracy in the boundary in one place can become a bigger problem later in the diagnostic or morphometric analysis if it is wrong. DS-HFN was the top-performing model on all four metrics. The outcomes substantiate its architectural conception, in particular, the dual-stream encoder’s proficiency in concurrently grasping the global context and localized detail, as well as the HyperFeature Embedding Module’s effectiveness in integrating multi-scale features. Additionally, the effects of the Gradient-Aligned Loss Function were visible not only in quantitative improvements but also qualitative ones. The changes in the boundary prediction are smoother, more anatomically logical, and less affected by noise (Figure 3).

**Figure 4:**
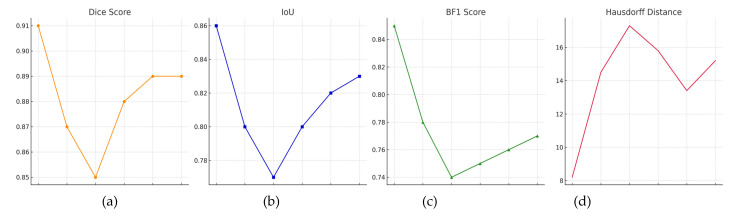
Ablation study results for the DS-HFN model. Each subplot illustrates the impact of removing specific architectural components on four evaluation metrics: (**a**) Dice Score, (**b**) Intersection over Union (IoU), (**c**) Boundary F1-Score, and (**d**) Hausdorff Distance. The full DS-HFN consistently outperforms all ablated variants, confirming the contribution of each module—dual-stream encoding, HyperFeature Embedding, boundary decoding, and Gradient-Aligned Loss—to overall segmentation accuracy and structural fidelity.

The results show that Deep Supervised HyperFusionNet (DS-HFN) is the best among the modern deep-learning algorithms for automatic detection and classification of histopathological nuclei in the breast cancer dataset (TNBC). The algorithm’s ability to adapt to specialized (TNBC) and more general (MoNuSeg) datasets indicates that it has good transfer learning properties, which further confirms its decent performance in clinical pathology pipelines where it is necessary to have the least number of errors, interpretability, and consistency. Ablation experiments involving turning off some of the architectural features or deactivating the loss function of the DS-HFN were designed to assess the contribution of individual parts to the final segmentation performance. These experiments were conducted using the TNBC validation folds while applying the same training settings, thus allowing for an exact comparison between the different model parts. This experimental setup makes it possible to identify which components are the main source of DS-HFN’s improved segmentation accuracy and contour fidelity. Initially, we ran some tests to check how much the dual-stream encoder architecture affects the outcome. We pit the full DS-HFN model against a version that employs a single-stream encoder, where only the local path (high-resolution features) was kept. The single-stream model showed a significant drop in Dice from 0.91 to 0.87, and its Boundary F1-Score decreased from 0.85 to 0.78, pointing out that the lack of global contextual features disoriented the model and made it unable to complete the object delineation task precisely, especially in those areas with clustered or ambiguous nuclear boundaries. On the other hand, a variant with only the global stream was not able to perform well in dense regions due to the lack of spatial resolution. Such a result backs up that the joint utilization of the global and local information through the dual encoder is crucial for reaching the best performance in various nuclear morphologies (Figure 4).

Thereafter, an attempt was made to test the HyperFeature Embedding Module (HFEM) contribution. The performance, as measured using all metrics, was significantly worse after the deletion of HFEM and the usage of simple additive fusion between streams. The Dice score went down to 0.88, and the F1 score of the IoU fell from 0.86 to 0.80. We can say that HFEM’s fusion by learning and dynamic reweighting of the multiscale features yields a more discriminative joint representation compared to a simple aggregation, if it is supported by these results. Qualitative assessment additionally showed that models without the HFEM module frequently did not recognize nuclei, which were either overlapped or had a complicated shape. This means that the module is the vital part to obtain details from the local scene and global context at the same time. To investigate the part that the dual-decoder design plays, we had the complete DS-HFN model and a simplified version that had a single decoder for the semantic segmentation and was without the boundary refinement branch. While this variant still performed well, the Boundary F1-Score went down from 0.85 to 0.76, and the Hausdorff Distance also rose from 8.2 to 13.4. The results provide evidence that directing and controlling flow on an edge-specific route improves both contour localization and the correction of structural details, particularly in the case of small or partially visible objects. At last, we assessed the extent of the Gradient-Aligned Loss Function LGLGA. If this loss term is excluded, then BF1 is reduced, and the Hausdorff Distance drastically increases to 15.2 pixels, which means that the misalignment between the predicted and the actual nuclear boundaries becomes great when LGLGA is removed. Even though the Dice Score stayed almost at the same level of 0.89, the breakdown in the contour part clearly shows the importance of LGLGA, which leads the model to correctly decide the segmentation of anatomical parts. The mixture of edge- and gradient-level supervision thus turned out to be more successful than region-only learning objectives. The quantitative summary of the ablation results is presented in Table 4.

**Figure 5:**
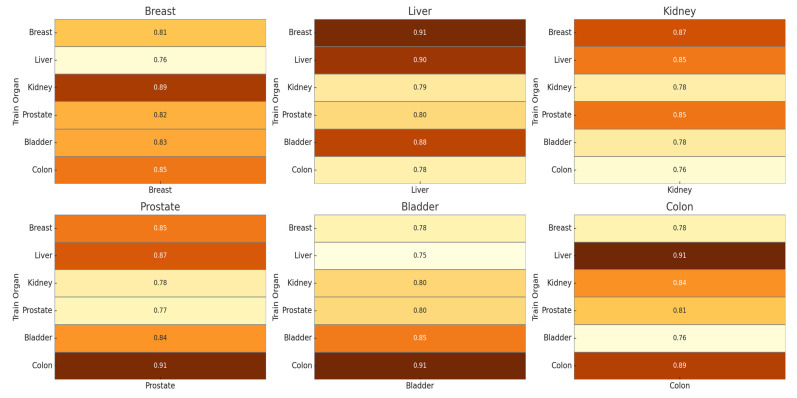
Organ-specific generalization heatmaps for DS-HFN. Each subplot illustrates the Dice Similarity Coefficient achieved when the model is tested on a fixed organ type (column) and trained on various other organs (rows). This layout highlights DS-HFN’s adaptability across domains and its robustness to inter-organ morphological variability within the MoNuSeg dataset.

The ablation study clearly shows that every element of the DS-HFN framework plays an important role in model performance. The dual-stream encoder and HFEM allow for deep, multi-scale feature learning, and the dual-decoder structure, along with the gradient-aligned supervision, helps to maintain the anatomical consistency at the boundary level. The collaboration of these parts is the main factor behind DS-HFN’s power to provide cutting-edge segmentation outcomes in tough histopathological datasets (Figure 5).

## 5. Conclusions

In this research, we presented a novel end-to-end deep learning architecture called DS-HFN for high-precision cell segmentation in histopathological images. The DS-HFN was tailored to tackle the main issues that come with overlapping nuclei, unclear edges, and cross-organ variabilities. It is a combinatorial assembly of several key architectural innovations: a dual-stream encoder that not only images the features of local parts but also the global context, a HyperFeature Embedding Module (HFEM) that distributes the changes of multiple scales adaptively, and a dual-decoder structure that concurrently performs semantic segmentation and boundary detail. To further push the model’s performance, the Gradient-Aligned Loss Function, which is designed explicitly for this model, helps it to stay aligned by providing the gradient of the predicted and the ground truth. The researchers performed a series of tests on two difficult datasets, namely TNBC and MoNuSeg. DS-HFN is shown to be significantly better than thirty state-of-the-art segmentation models across numerous evaluation metrics, including Dice Similarity, Intersection over Union, Boundary F1-Score, and Hausdorff Distance. In particular, DS-HFN outperforms these methods not only in dense and irregular nuclear regions but also in the unrepresented organs during training, indicating robustness and generalization ability. Ablation analyses also provided the root to each architectural part. The study shows that the equal impact of multiple encoding, adaptive fusion, and boundary-aware decoding is essential for attaining the current state-of-the-art performance. The DS-HFN framework aims at anatomically precise and clinically relevant computational pathology tools that have come of age. Being able to reason at both the region occupancy and structural fidelity levels makes it particularly powerful in downstream tasks, such as morphometric analysis, cellular phenotyping, and cancer grading. Additionally, its modularity and fast inference make it a potential candidate for incorporation into digital pathology workflows. Furthermore, extending the model for instance-level segmentation of nuclei and adapting the dual-stream fusion paradigm to 3D volumetric data in whole-slide imaging may be the direction of the future. It is also possible that domain adaptation methods and semi-supervised learning strategies will be incorporated in the model, thereby facilitating utilization in low-resource clinical settings without much-grained annotations. DS-HFN is a single, efficient, and deeply accurate solution to the long-standing histopathological nuclei segmentation problem. Its performance, generalizability, and architectural novelty collectively contribute to the progress of intelligent digital pathology and shed new light on medically robust AI systems in medical imaging.

## Figures and Tables

**Table 1:** Dataset detailed information.

Dataset	Tissue Source(s)	Images	Annotation Type	Image Size	Challenges
TNBC	Breast (TNBC)	50	Binary masks	512 × 512	Dense overlap, jagged contours
MoNuSeg	Multi-organ	30	Instance masks	~2000 × 2000	Cross-domain variability, scale heterogeneity

**Table 2:** Summary of DS-HFN training configuration.

Parameter	Value/Setting
Framework	PyTorch v2.1.0 (CUDA 11.8)
Optimizer	AdamW
Initial Learning Rate	1 × 10^−4^
Learning Rate Schedule	Cosine annealing with warm restarts
Weight Decay	0.01
Batch Size	16
Epochs	200
Early Stopping Patience	15 epochs
Mixed Precision Training	Enabled (PyTorch AMP)
Gradient Clipping Norm	5.0
Convolution Initialization	He initialization
Input Size	256 × 256
Image Preprocessing	Grayscale conversion, standardization
Data Augmentation	Rotation, flipping, elastic deformation
Random Seed	42
Prediction Threshold	0.5
Loss Function Weights (α/β/γ)	0.6/0.2/0.2
Checkpointing Criterion	Best validation Dice score
Edge Enhancement Method	Sobel filter (3 × 3 kernel)
Train–Validation–Test Split	5-fold cross-validation with stratified sampling

**Table 3:** Comparison results with SOTA models.

Model	Dice Score	IoU	BF1 Score	Hausdorff Distance
DS-HFN (ours)	0.91	0.86	0.85	8.2
Xu et al. [1]	0.89	0.738	0.68	13.69
Chen et al. [4]	0.89	0.793	0.729	10
Guan et al. [5]	0.89	0.788	0.736	16.18
Liu et al. [18]	0.873	0.702	0.79	16.04
Hasan et al. [20]	0.869	0.779	0.685	12.19
Kadaskar et al. [21]	0.866	0.788	0.708	14.06
Qian et al. [22]	0.865	0.756	0.716	15.39
Jaafar et al. [25]	0.861	0.768	0.756	10.33
Cao et al. [26]	0.859	0.822	0.725	15.7
Ding et al. [28]	0.857	0.775	0.738	13.63
Sufyan et al. [32]	0.853	0.817	0.715	16.21
Cao et al. [33]	0.852	0.753	0.8	15.24
Sreekumar et al. [35]	0.846	0.84	0.731	18.87
Xu et al. [1]	0.843	0.765	0.758	14.89
Imtiaz et al. [41]	0.882	0.801	0.791	11.6
Lin et al. [42]	0.871	0.783	0.764	13.8

**Table 4:** Ablation study of DS-HFN components (on TNBC dataset).

Variant	Dice Score	IoU	BF1 Score	Hausdorff Distance
Full DS-HFN (proposed)	0.91	0.86	0.85	8.2
w/o Global Stream (Local Only)	0.87	0.80	0.78	14.5
w/o Local Stream (Global Only)	0.85	0.77	0.74	17.3
w/o HFEM Fusion	0.88	0.80	0.75	15.8
w/o Boundary Decoder	0.89	0.82	0.76	13.4
w/o Gradient-Aligned Loss LGLGA	0.89	0.83	0.77	15.2

## Data Availability

All used dataset are available online and are open accessed.

## References

[1] Xu J., Shi L., Li S., Zhang Y., Zhao G., Shi Y., Li J., Gao Y. (2025). PointFormer: Keypoint-Guided Transformer for Simultaneous Nuclei Segmentation and Classification in Multi-Tissue Histology Images. IEEE Trans. Image Process..

[2] Murmu A., Kumar P. (2025). Automated breast nuclei feature extraction for segmentation in histopathology images using Deep-CNN-based gaussian mixture model and color optimization technique. Multimed. Tools Appl..

[3] Pons S., Dura E., Domingo J., Martin S. (2025). Advancing histopathology in Health 4.0: Enhanced cell nuclei detection using deep learning and analytic classifiers. Comput. Stand. Interfaces.

[4] Chen J., Wang R., Dong W., He H., Wang S. (2025). HistoNeXt: Dual-mechanism feature pyramid network for cell nuclear segmentation and classification. BMC Med. Imaging.

[5] Guan B., Chu G., Wang Z., Li J., Yi B. (2025). Instance-level semantic segmentation of nuclei based on multimodal structure encoding. BMC Bioinform..

[6] Abdusalomov A., Umirzakova S., Boymatov E., Zaripova D., Kamalov S., Temirov Z., Jeong W., Choi H., Whangbo T.K. (2025). A Human-Centric, Uncertainty-Aware Event-Fused AI Network for Robust Face Recognition in Adverse Conditions. Appl. Sci..

[7] Zhang X., Zhang Z.H., Liu Y.M., Zhao S.L., Zhao X.T., Zhang L.Z., Gu C.D., Zhao Y. (2025). Investigating lung cancer microenvironment from cell segmentation of pathological image and its application in prognostic stratification. Sci. Rep..

[8] Lakshmi Priya C.V., Biju V.G., Bhooshan R.S. (2025). Enhancing nuclei segmentation in breast histopathology images using U-Net with backbone architectures. Comput. Biol. Med..

[9] Bakhtiyorov S., Umirzakova S., Musaev M., Abdusalomov A., Whangbo T.K. (2025). Real-Time Object Detector for Medical Diagnostics (RTMDet): A High-Performance Deep Learning Model for Brain Tumor Diagnosis. Bioengineering.

[10] Sunesh Tripathi J., Saini A., Tiwari S., Kumari S., Taqui S.N., Almoallim H.S., Alharbi S.A., Raghavan S.S. (2025). Nucleus segmentation from the histopathological images of liver cancer through an efficient deep learning framework. Multimed. Tools Appl..

[11] Yao Y., Hu Y., Xue Y., Li S., Huang J., Wang H., He J. (2025). UPHGAN: Generative Adversarial Network Based on Unet512 and PatchGAN Fusion with Huber Loss Function for Immunohistochemical Cell Nucleus Segmentation. International Conference on Neural Information Processing.

[12] Prabhu S., Prasad K., Robels-Kelly A., Lu X. (2022). AI-based carcinoma detection and classification using histopathological images: A systematic review. Comput. Biol. Med..

[13] Dumbhare P., Dubey Y., Phuse V., Jamthikar A., Padole H., Gupta D. (2022). November. Combining deep-learned and hand-crafted features for segmentation, classification and counting of colon nuclei in H &E Stained histology images. Proceedings of the International Conference on Computer Vision and Image Processing.

[14] Abdusalomov A., Mirzakhalilov S., Umirzakova S., Kalandarov I., Mirzaaxmedov D., Meliboev A., Cho Y.I. (2025). Optimized Lightweight Architecture for Coronary Artery Disease Classification in Medical Imaging. Diagnostics.

[15] Lou W., Wan X., Li G., Lou X., Li C., Gao F., Li H. (2024). Structure embedded nucleus classification for histopathology images. IEEE Trans. Med. Imaging.

[16] Hoque M.Z., Keskinarkaus A., Nyberg P., Seppänen T. (2024). Stain normalization methods for histopathology image analysis: A comprehensive review and experimental comparison. Inf. Fusion.

[17] Ramakrishnan V., Artinger A., Daza Barragan L.A., Daza J., Winter L., Niedermair T., Itzel T., Arbelaez P., Teufel A., Cotarelo C.L. (2024). Nuclei Detection and Segmentation of Histopathological Images Using a Feature Pyramidal Network Variant of a Mask R-CNN. Bioengineering.

[18] Liu A., Zhang Y., Xia Y., Wan X., Zhou L., Song W., Zhu S., Yuan X. (2024). Classes U-Net: A method for nuclei segmentation of photoacoustic histology imaging based on information entropy image classification. Biomed. Signal Process. Control.

[19] Zheng K., Pan J., Jia Z., Xiao S., Tao W., Zhang D., Li Q., Pan L. (2024). A method of nucleus image segmentation and counting based on TC-UNet++ and distance watershed. Med. Eng. Phys..

[20] Hasan M.J., Ahmad W.S.H.M.W., Fauzi M.F.A., Lee J.T.H., Khor S.Y., Looi L.M., Abas F.S. (2025). An Attention Based Model for Histopathology Image Nuclei Segmentation. Proceedings of the 2025 IEEE 22nd International Symposium on Biomedical Imaging (ISBI).

[21] Kadaskar M., Patil N. (2024). ANet: Nuclei Instance Segmentation and Classification with Attention-Based Network. SN Comput. Sci..

[22] Qian Z., Wang Z., Zhang X., Wei B., Lai M., Shou J., Fan Y., Xu Y. (2024). MSNSegNet: Attention-based multi-shape nuclei instance segmentation in histopathology images. Med. Biol. Eng. Comput..

[23] Chen F., Liu H., Zeng Z., Zhou X., Tan X. (2022). BES-Net: Boundary Enhancing Semantic Context Network for High-Resolution Image Semantic Segmentation. Remote Sens..

[24] Zhao Z., Chen H., Li J., Wang L. Boundary Attention U-Net for Kidney and Kidney Tumor Segmentation. Proceedings of the 2022 44th Annual International Conference of the IEEE Engineering in Medicine & Biology Society (EMBC).

[25] Jaafar R., Yazid H., Farhat W., Amara N.E.B. (2025). SBC-UNet3+: Classification of Nuclei in Histology Imaging Based on Multi Branch UNET3+ Segmentation Model. Proc. Copyr..

[26] Cao R., Meng Q., Tan D., Wei P., Ding Y., Zheng C. (2024). AER-Net: Attention-Enhanced Residual Refinement Network for Nuclei Segmentation and Classification in Histology Images. Sensors.

[27] Chen Z.M., Liao Y., Zhou X., Yu W., Zhang G., Ge Y., Ke T., Shi K. (2024). Pancreatic cancer pathology image segmentation with channel and spatial long-range dependencies. Comput. Biol. Med..

[28] Ding R., Zhou X., Tan D., Su Y., Jiang C., Yu G., Zheng C. (2024). A deep multi-branch attention model for histopathological breast cancer image classification. Complex Intell. Syst..

[29] Graham S., Vu Q.D., Raza S.E.A., Azam A., Tsang Y.W., Kwak J.T., Rajpoot N. (2019). Hover-net: Simultaneous segmentation and classification of nuclei in multi-tissue histology images. Med. Image Anal..

[30] Chen H., Qi X., Yu L., Heng P.A. DCAN: Deep contour-aware networks for accurate gland segmentation. Proceedings of the IEEE conference on Computer Vision and Pattern Recognition.

[31] Zhou Y., Onder O.F., Dou Q., Tsougenis E., Chen H., Heng P.A., Chung A., Gee J., Yushkevich P., Bao S. (2019). CIA-Net: Robust Nuclei Instance Segmentation with Contour-Aware Information Aggregation. Information Processing in Medical Imaging.

[32] Sufyan A., Fauzi M.F.A., Kuan W.L. (2025). M3-Net: A Multi-Scale Nuclei Segmentation Model for Breast Cancer Histopathology Using Contextual Patches and Attention Mechanism. Proceedings of the 2025 IEEE 22nd International Symposium on Biomedical Imaging (ISBI).

[33] Cao L., Pan K., Ren Y., Lu R., Zhang J. (2024). Multi-branch spectral channel attention network for breast cancer histopathology image classification. Electronics.

[34] Xu C., Huang S., Zhang Y., Hu D., Sun Y., Li G. (2025). DualBranch-FusionNet: A Hybrid CNN-Transformer Architecture for Cervical Cell Image Classification. Int. J. Imaging Syst. Technol..

[35] Sreekumar S.P., Palanisamy R., Swaminathan R. (2024). An Approach to Segment Nuclei and Cytoplasm in Lung Cancer Brightfield Images Using Hybrid Swin-Unet Transformer. J. Med. Biol. Eng..

[36] Yıldız S., Memiş A., Varlı S. (2024). Segmentation of Cell Nuclei in Histology Images with Vision Transformer Based U-Net Models. Proceedings of the 2024 32nd Signal Processing and Communications Applications Conference (SIU).

[37] Mezei T., Kolcsár M., Joó A., Gurzu S. (2024). Image Analysis in Histopathology and Cytopathology: From Early Days to Current Perspectives. J. Imaging.

[38] Han K., Wang Y., Guo J., Tang Y., Wu E. (2022). Vision gnn: An image is worth graph of nodes. Adv. Neural Inf. Process. Syst..

[39] Tan C.W., Yu P.D., Chen S., Poor H.V. (2025). DeepTrace: Learning to optimize contact tracing in epidemic networks with graph neural networks. IEEE Trans. Signal Inf. Process. Over Netw..

[40] Qin J., Liu J., Liu W., Chen H., Zhong D. (2024). MATNet: A multi-attention transformer network for nuclei segmentation in thymoma histopathology images. Multimed. Tools Appl..

[41] Imtiaz T., Fattah S.A., Kung S.Y. (2023). BAWGNet: Boundary aware wavelet guided network for the nuclei segmentation in histopathology images. Comput. Biol. Med..

[42] Lin Y., Qu Z., Chen H., Gao Z., Li Y., Xia L., Ma K., Zheng Y., Cheng K.T. (2023). Nuclei segmentation with point annotations from pathology images via self-supervised learning and co-training. Med. Image Anal..

